# Effect of the buccal fat pad in the prevention of zygomatic implant surgery postoperative complications: A pilot study

**DOI:** 10.4317/medoral.25792

**Published:** 2023-06-18

**Authors:** Sonia Blanco-Ruiz, Pedro Molinero-Mourelle, Miriam Blanco-Ruiz, Francisco González Fernández-Tresguerres, Senén Blanco-Samper, Juan López-Quiles

**Affiliations:** 1 Department of Dental Clinical Specialties, Faculty of Dentistry, Complutense University of Madrid, Madrid, Spain; 2 Department of Conservative Dentistry and Orofacial Prosthodontics, Complutense University of Madrid, Madrid, Spain; 3 Department of Reconstructive Dentistry and Gerodontology, University of Bern, Bern, Switzerland; 4 Department of Otorhinolaryngology, Head and Neck Surgery, University General Hospital, Ciudad Real, Spain

## Abstract

**Background:**

Zygomatic implants have been used to treat severe atrophy maxilla. Since its description, the technique has been improved in order to reduce patient morbidity as well as prosthesis rehabilitation time. Despite the improvements in the procedure, zygomatic implant treatments still have complications related to the peri-implant soft-tissue; a probing depth greater than 6 millimeter (mm) and a prevalence of bleeding on probing of 45% have been described. The mobilization of the buccal fat has been used to manage different oral and maxillofacial soft-tissue pathologies. The aim of this study was to assess whether the buccal fat pad might prevent mucosal dehiscence and avoid potential postoperative complications when is placed covering the body part of the zygomatic implants.

**Material and Methods:**

In this pilot study, 7 patients were enrolled and a total of 28 zygomatic implants were placed and evaluated during a 12-month follow-up period. Surgical sites were randomly divided into two groups before implant placement: control group (A; in which no buccal fat pad was applied) and experimental group (B). Peri-implant soft tissue thickness difference, pain using a Visual Analog Scale (VAS), swelling, hematoma, buccal soft tissue healing and sinusitis, were evaluated. The implant survival rate was determined according the Aparicio success criteria and compared between the control and experimental procedure.

**Results:**

A nonstatistical difference was found between groups regarding to pain. The experimental group showed higher soft-tissues thickness (*p*= 0.03) and the implant survival rate was 100% in both groups.

**Conclusions:**

The mobilization of the buccal fat pad to cover the body of the zygomatic implants increases peri-implant soft-tissue thickness, without increasing the postoperative pain.

** Key words:**Zygomatic implants, maxillary atrophy, Complications, Postoperative care, Swelling, Pain.

## Introduction

Complex oral surgeries with the use of bone regeneration materials have been used to treat bone reabsorption processes (1). However, the management of severe maxillary atrophy in long-term edentulous patients is still a challenge in dentistry.

Since the first description of zygomatic implants and the surgery procedure in 1990 (2), the technique has been modified and improved (2-6) to reduce patient morbidity as well as treatment cost and time (7,8). Some authors have pointed out that survival rates of zygomatic implants are similar to conventional implants rates, being above 99.5% (8,9). Nevertheless, zygomatic implants are not devoid of complications, (5,7-14) the most frequent being peri-implant soft-tissue disruptions, with a mean probing depth greater than 6 millimeter (mm) and a prevalence of bleeding on probing of 45%.

Although soft-tissue variations have been related to the presence of pathogenic bacteria, probing depth was found to be increased in these implants even in the absence of microorganisms (14). In connection with this, peri-implant inflammation followed by soft-tissue recessions in extrasinusal zygomatic implants could potentially derive from the exposed part of the implant because the zygomatic implant body is in intimate contact with oral mucosal tissue (5).

The buccal fat pad has been used to manage diverse sort of oral and maxillofacial complications (15,16). Some authors have employed the buccal fat pad during zygomatic implant surgeries and have suggested the absence of post-surgery and implant complications during a follow-up period of more than 15 months (12).

The aim of the study was to assess if the repositioning of the buccal fat pad might prevent the gingival recession. Hence, this study aimed to test hypothesis that covering the exposed part of the zygomatic implant with the buccal fat pad would prevent future recession of peri-implant soft-tissues because the fatty-tissue might increase oral mucosal thickness.

## Material and Methods

- Study design

A pilot, prospective, single-blind, split-mouth and randomized clinical trial was conducted in patients who required treatment with two implants in each zygomatic bone. Participants were followed up at 72 hours, 7 days, 15 days, 3-, 6- and 12-months post-surgery; The present trial was conducted in accordance with Helsinki Declaration (2013) and the Ethical committee approvement from Clínico San Carlos Hospital (19/531-R_P).

- Participants

Participants were recruited through the research project carried out by the Oral Surgery postgraduate clinic at Universidad Complutense Madrid. All patients enrolled were over 18 years-old, with an ASA I or ASA II surgical risk according to the American Society of Anesthesiologists (ASA). Moreover, patients who had less than 4 mm of residual maxillary bone measured with cone-beam computer tomography needed treatment with two zygomatic implants in each malar bone. All participants were informed in detail about the nature of the study and gave their written informed consent before being included.

- Surgical procedure

**Figure 1:**
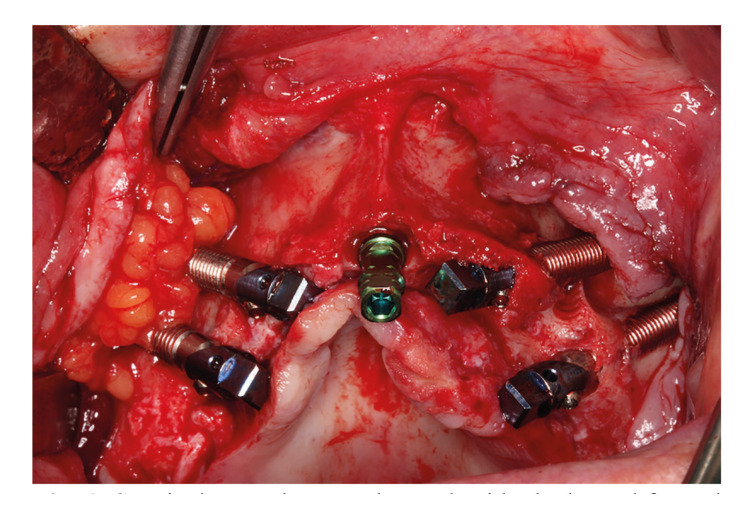
Surgical procedure. In the study side the buccal fat pad is covering body implants.

Patients were treated under intravenous conscious sedation with benzodiazepine (Midazolam 1 mg/ml, Anesfarma, Spain) combined with local anesthesia of articaine hydrochloride with epinephrine 0,1 mg/ milliliter(ml) (Ultracain 40 mg, Normon, Spain). The surgical procedure was performed by a highly-experienced maxillofacial surgeon with over 30 years of clinical experience (J.L-Q.). All zygomatic implants were placed in the patient in only one surgical session. Moreover, each participant was part of two study groups: the control group (A) in which after two zygomatic implants, the covering flap was sutured and the experimental group (B) in which, after implant placement, the buccal fat pad was repositioned covering the out-bone implant body, (Fig. 1) followed by the flap suture.

All previous surgery steps were the same for both groups and the surgeon placed HE Zygomatic Implant Neodent® (Straumann AG; Basel, Switzerland) following its drill sequence system. After all zygomatic implants were placed, the implants were classified in accordance with ZAGA classification system (6). Postoperative drug treatment included omeprazole 20 mg (Kern Pharma; Madrid, Spain) once-daily for 20 days, Augmentin 875/125 mg (GSK, GlaxoSmithKline; Brentford, U.K) 1 dose every 8 hours for 15 days, Deflazacort 30m (Laboratorios Menarini S.A; Napoles, Italy) 2 doses/24 hours for 5 days, Dexketoprofen 25 mg (Laboratorios Menarini S.A; Napoles, Italy) 1 dose/8 hours for 5 days, Metamizole 575 mg (Laboratorio Stada S.L; Barcelona, Spain) 1 dose/8 hours for 3 days. Treatment also included: oxymetazoline 0.5 mg/ml nasal spray, a nostril-nebulizer three times a day for 3 days, fluticasone furoate, two spray actuations daily for 15 days and nasal wash with a saline solution (GSK, GlaxoSmithKline; Brentford, U.K).

- Outcome measures

**Figure 2:**
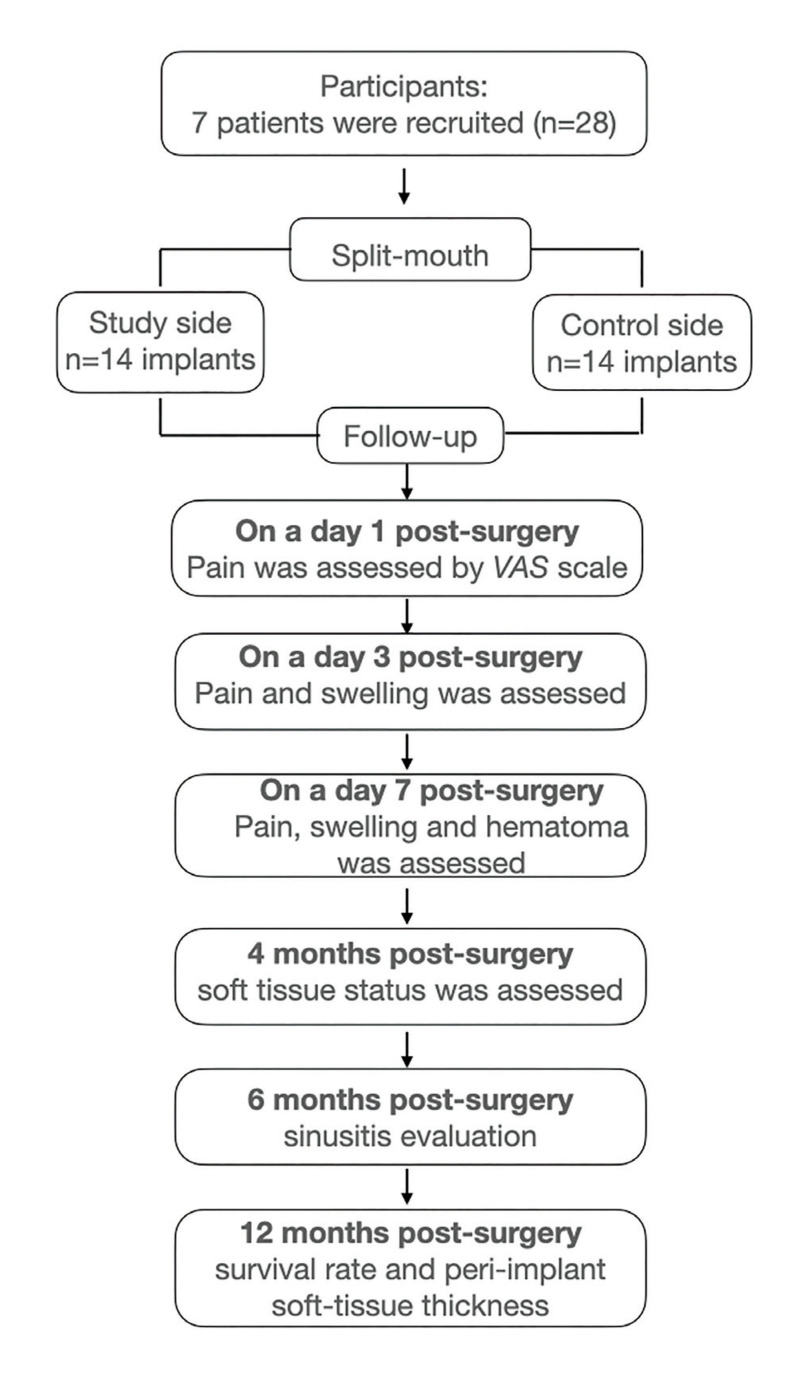
Flow chart of the study protocol according to Consort guidelines, including the number of participants enrolled and the period of the outcome measures.

The primary outcome was peri-implant soft tissue thickness difference between both groups, using the average of three measurement points on the buccal side of the implant platform registered with a periodontal probe (HH12 periodontal probe, Deppeler SA). Secondary outcomes were: pain assessed using a Visual Analog Scale (VAS), swelling assessed using the Amin & Laskin method (17), hematoma measured according to the Gutiérrez y Wuesthoff method (18). Sinusitis was evaluated using the Hwang questionnaire (19) and the implant survival rate following Aparicio success criteria (20). All measured outcomes variables were assessed by an independent, trained and non-blinded researcher (S.B.R) (Fig. 2).

- Sample size, randomized sequence, and statistical analysis

Being a pilot study, no sample size was calculated, and seven patients were enrolled in this study. Hence, a total of 28 zygomatic implants were analyzed been it considered the statistical unit.

The sample was randomized through a simple process using Matlab 28b (Math Works. Natick, Massachusetts, USA). A descriptive statistical analysis was carried out using measures of central and dispersion tendency and percentages. In addition, Shapiro-wilk test was used to assess the normal distribution of the data and a non-parametrical test (Wilcoxon) to contrast hypothesis.

## Results

After 12 months of follow-up, 28 zygomatic implants were evaluated in seven patients (71% female and 29% male). The mean age among participants was 55.57±6.39 years-old, the average of the surgical time spent was 172.14±54.60 minutes (Table 1).

Shapiro-wilk test confirmed the normal distribution of pain data in both groups; however, swelling parameter distribution did not fit the standardized criteria, so both clinical outcomes were assessed with a non-parametric test (Wilcoxon).

Regarding to the swelling outcome variable, a statistical difference was found between both groups (*p*= 0.043). Indeed, the mean percentage of swelling in the study group was 12.16%, compared to 8.05% in the control group. Moreover, the maximum percentage of swelling registered in the experimental group was a 6.79% greater than in the control-group, with a maximum proportion of swelling of 22.42% and 15.63%, respectively. Swelling and pain outcomes are displayed in Table 2.

Three patients had hematoma that happened always as a bilateral episode. However, a greater number of faces-areas were affected in the study group. Moreover, cheek section was drawn in all test hemifaces sides while only that region was just affected in one out of three control group. In a control-side, the suture dehiscence occurred at day 5 post-surgery in one participant (14,28%). On the other hand, six months after treatment, one patient in the control-group showed sinusitis and skin periorbital fistula.

Peri-implant soft-tissue thickness was evaluated in 20 out of 28 implants due to two patients did not attend to the follow-up, the experimental group showed an average of 5.2±1.47 mm while in the control group it was 3±1.05 mm, (*p*=0.03). In the control group a 14.3 % of the implant showed 10 mm of mucosal dehiscence however no one of the implant from the study group. This showed in Table 3.

**Table 1:**
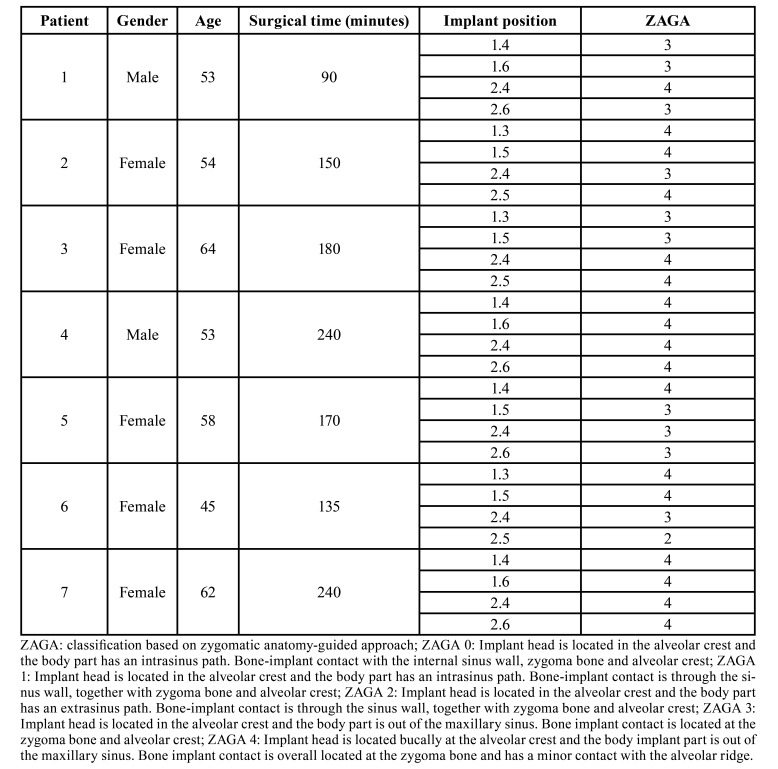
Descriptive data about demographical parameters, surgical time, implant position and type.

**Table 2:**
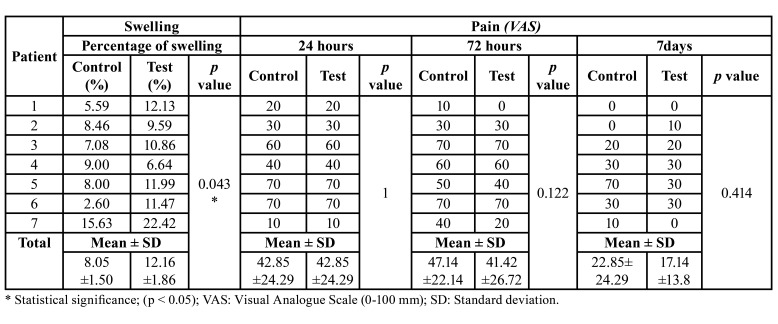
Comparison of two outcomes between groups (control-test): the percentage of swelling and pain (VAS) in each participant and the mean value together with the statistical deviation.

**Table 3:**
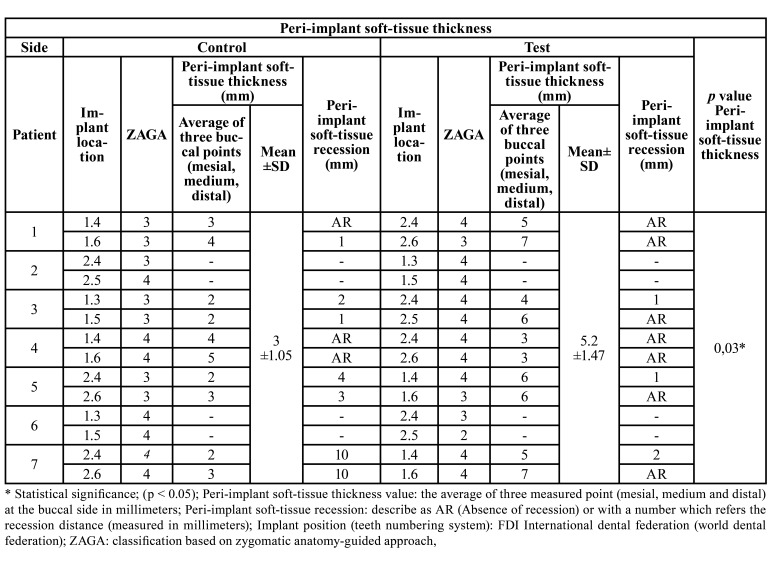
Peri-implant soft-tissue thickness analysis and description of peri-implant soft-tissue recession.

## Discussion

Zygomatic implant surgery is considered an advanced surgical intervention and requires more surgical time than conventional implant techniques. However, the development and variations in zygomatic implant placement technique have reduced the surgical time (5) overall, buccal fat pad replacement involves more surgical trauma because an additional procedure is needed. Considering the literature, Guennal, *et al*. reported that patients showed an overall worse postoperative period when buccal fat pad had been mobilized (12). In the same line, the present study showed higher swelling rates and more areas affected by hematoma. However, non-significant differences in pain were found between groups.

On the other hand, Esposito, *et al*., linked bruise occurrence with the use of rotary drill material during zygomatic implants placement (21). Therefore, the presence of bilateral hematoma might be related with the drill material instead of the mobilization of the buccal fat pad even though a greater area was affected in the experimental group.

Extrasinusal zygomatic implants enhance prothesis rehabilitation, shrinking cantilevers palatal-buccal owing to implant head arise at bone crest level (5,6,10). Nevertheless, the intimated contact between extrasinus implants body with buccal soft tissue may alter them (5,12). Molinero-Mourelle, *et al*. and Charcanovic, *et al*. agree with the fact that buccal mucosal modification is the third more frequent complication (8,11). Moreover, Yates, *et al*., found gingival recession from between 2-4 mm in zygomatic implants after a follow-up period of 6 years (13).

Conventional implants with a thin soft-tissue phenotypes, required subepithelial or free connective tissue graft to prevent the development of peri-implant mucosal dehiscence (22,23). In addition, based on current knowledge, the buccal fat pad has been successfully used to cover tooth recessions (24,25). Hence, from other perspective but following with a similar investigation line, the present study showed that peri-implant buccal mucosal dehiscence was significantly worse in those implants that were not covered with the buccal fat pad. Also, we found higher peri-implant soft tissue thickness in the study group compared to control group, the average of the peri-implant soft-tissue thickness being more than 2 mm thicker in the study side as compared to the control side. Therefore, the mobilization of the buccal fat pad might prevent peri-implant recessions.

Regarding other assessed complications, such as sinusitis (8,10,11,26,27), this study agrees with other authors in that extrasinusal zygomatic implant show lower incidence of sinusitis than intrasinusal zygomatic implants (10,28).

The survival rate of the zygomatic implants would be greater than 97% (28-30) and 95% (27) after a follow-up period of 10 years and 18 years, respectively. This study did not show any failure of the zygomatic implants during a follow-up period of one year.

The authors of the present study acknowledge some limitations in the study design (pilot study and single blind) that could had impacted in the study results. It must be taken into consideration that the person who gathered the outcomes variables was not blinded. In addition, not all implants were classified as the same type of ZAGA; future studies should homogenize and group patients regarding the ZAGA classification. Moreover, there were more female patients, and the sample size could not be representative. Yet, even though we cannot establish accurate conclusions about the use of the buccal fat pad in the prevention of extrasinus zygomatic implants recessions due to the paucity of the sample size (external validity), this study showed promising outcomes. Therefore, more randomized clinical trials are warranted to confirm the technique effectiveness in complications in zygomatic implants.

## Conclusions

Within the limitations of this pilot study, the use of the buccal fat pad for covering the body part of the zygomatic implant appeared to be a reliable option in the prevention of peri-implant soft tissue dehiscence, as the buccal fat pad increased the soft-tissue around zygomatic implants in the study group. However, this procedure was associated with an increased rate of postoperative swelling.
